# Mobile genetic elements shape the evolution and adaptation of the marine *Sulfitobacter* genus

**DOI:** 10.1128/msystems.00479-26

**Published:** 2026-06-15

**Authors:** Mustafa Guzel, Frank May, Alison Buchan

**Affiliations:** 1 Department of Microbiology, University of Tennessee 189504, Knoxville, Tennessee, USA; 2 Department of Food Engineering, Hitit University, Corum, Türkiye; University of Florida, Gainesville, Florida, USA

**Keywords:** *Roseobacteraceae*, *Sulfitobacter*, mobile genetic element, plasmid, horizontal gene transfer

## Abstract

Mobile genetic elements (MGEs) are essential for facilitating horizontal gene transfer and play crucial roles in the evolution and adaptive capabilities of bacterial species. Here, we analyzed closed genomes from the marine *Sulfitobacter* genus to assess plasmid contributions to ecological adaptability and evolutionary diversification. Our analysis of 153 *Sulfitobacter* plasmids from 36 strains representing 8 species shows extensive plasmid conservation within species (e.g., >95% nucleotide identity for flagellar plasmids) alongside significant mosaicism across 60% of plasmids. Insertion sequences (IS) elements are nearly ninefold more concentrated on plasmids relative to chromosomes, suggestive of active genetic exchange in this replicon class. Network analysis identified 14 primary plasmid clusters, with species-specific conservation patterns and evidence of inter-species gene transfer. In *Sulfitobacter pontiacus* strain CB2047, we discovered chromosomal integration of a 280 kb plasmid encoding a toxin-antitoxin system, *rrn* operon, as well as a chromosomal partitioning system. These findings demonstrate that plasmids function as key drivers of evolution and adaptation in *Sulfitobacter,* serving as both repositories of conserved adaptive traits and platforms for ongoing genetic innovation.

**IMPORTANCE:**

Plasmids are increasingly recognized as crucial drivers of bacterial evolution and adaptation, yet their roles in shaping marine microbial communities are poorly understood. Here, we provide a comprehensive analysis of plasmid diversity and evolution within *Sulfitobacter*, a broadly distributed and metabolically versatile marine bacterial genus, in which ~15% of genome content is plasmid-encoded. We propose that *Sulfitobacter* plasmids serve dual evolutionary roles: maintaining highly conserved species-specific traits essential for survival (such as flagellar motility and biofilm formation), while simultaneously functioning as platforms for genetic innovation through extensive horizontal gene transfer. The discovery of a large plasmid integrated into the chromosome of one strain highlights that episomal elements can transition to stable chromosomal inheritance in this genus. These findings advance our understanding of how marine bacteria balance genomic stability with adaptive flexibility, providing insights applicable to microbial evolution in dynamic ocean environments.

## INTRODUCTION

Mobile genetic elements (MGEs) are dynamic segments of DNA with the remarkable ability to move within and across genomes. These elements are pivotal in facilitating horizontal gene transfer, significantly influencing the evolution and adaptive capabilities of bacterial species. Notably, MGEs, particularly conjugative plasmids, enhance bacterial fitness by carrying and disseminating adaptive traits, including genes that confer antibiotic and heavy metal resistance, virulence factors, anti-phage defense systems, and a capacity to transform or metabolize specific organic compounds, providing host cells with competitive advantages across diverse environments (1, 2).

The *Roseobacteraceae* family of marine bacteria represents an abundant and active lineage that exhibits a complex evolutionary history heavily influenced by MGEs (3). *Roseobacteraceae* are metabolically versatile and genetically diverse (3). Recent studies have highlighted the polyphyletic nature of this lineage, suggesting that rather than originating from a single common ancestor, it has diversified through repeated episodes of genome expansion and extensive horizontal gene exchange (3, 4). A key driver of this polyphyly is the activity of MGEs, including plasmids, transposons, and prophages, which have facilitated extensive horizontal gene transfer across members.

The genomic diversity of the *Roseobacteraceae* reveals evolutionary adaptations to marine environments, affecting their metabolic pathways and ecological roles (5). The adaptive versatility of *Roseobacteraceae* members is regularly driven by plasmids, as these MGEs contribute to their niche-specific metabolic capabilities (6). Among *Roseobacteraceae* family members, numerous species encode lifestyle-determining genes on their plasmids. Gene clusters enabling flagellar motility and biofilm enhancement (e.g., rhamnose biosynthesis) are commonly found on plasmids in various *Roseobacteraceae* genera (7). Consequently, plasmid loss can lead to significant alterations in cellular physiology, including loss of motility, alterations in biofilm formation capabilities, and stabilization of chromosomally encoded prophages (8, 9). Plasmids of the *Roseobacteraceae* are also known to mediate a range of host metabolic abilities, exemplified through plasmid-encoded anoxygenic photosynthesis gene clusters that facilitate photoheterotrophic capabilities (10). Additionally, *Roseobacteraceae* plasmids have been proposed to influence chromosomal gene expression, an observation believed to result from plasmid-encoded regulatory elements, such as alternative sigma factors (11). Together, these plasmid-encoded traits demonstrate the importance of MGEs in influencing the ecological adaptability and niche-specific fitness of their hosts (9).

In the context of plasmid-mediated gene transfer within *Roseobacteraceae*, it is pertinent to distinguish between conjugative, mobilizable, and non-mobilizable plasmids, as all are prevalent in this bacterial family. Conjugative plasmids are self-transmissible and encode all the necessary machinery for plasmid transfer via conjugation, including the type IV secretion system (T4SS) (12). In contrast, mobilizable plasmids canonically lack the T4SS and rather carry a mobilization (*mob*) gene. In *Roseobacteraceae*, *mobQ*-type relaxases are found in mobilizable plasmids. Even in the absence of conjugation machinery on the same element, these *mobQ*-type relaxases enable plasmid transfer amongst family members (6). It is hypothesized that this transfer is facilitated by a conjugative plasmid-encoded T4SS present in the same cell, although this has yet to be conclusively demonstrated in *Roseobacteraceae* (13). As the name implies, non-mobilizable plasmids are those that are not subject to transfer between cells (14) and are defined as those lacking recognized mobility genes. Recent experimental work has demonstrated that large, conjugative plasmids from *Dinoroseobacter shibae* can be transferred to diverse representatives of the *Roseobacteraceae*, underscoring the role of plasmids in facilitating genetic exchanges and driving evolutionary changes across genera within this family (15, 16). Indeed, observation of identical plasmids in phylogenetically and geographically distant *Roseobacteraceae* representatives supports these findings and highlights the contribution of plasmids to evolution and niche adaptation of group members (17).

Here, we consider the role of plasmids in the evolution of *Sulfitobacter*, an environmentally abundant and active genus of the *Roseobacteraceae* family. Our analyses highlight the abundance, diversity, and conservation of plasmids across different *Sulfitobacter* species. We identify extensive plasmid conservation within, but diversity across, species. We also provide evidence of chromosomal integration of a large plasmid in one strain (*Sulfitobacter pontiacus* CB2047).

## RESULTS

### Genomic landscape and phylogenetic framework

To investigate the role of plasmids in the evolution of *Sulfitobacter*, we performed a stepwise analysis focusing on plasmid diversity, conservation, and evidence for recombination. We first assessed the genomic landscape of *Sulfitobacter* species to establish phylogenetic relationships and core genome composition. Following an initial screen of 374 *Sulfitobacter* genomes (Table S2), consisting of 27 characterized *Sulfitobacter* species (as defined by NCBI [Table S1]), we reconstructed genus-level evolutionary relationships from a dereplicated 129-genome set, representing 22 species (Fig. S1). To identify plasmid contributions to *Sulfitobacter* species’ genetic diversity, subsequent analyses exclusively focused on 36 closed genomes for which confirmation of complete plasmid sequences is highest.

The 36-member closed genome set represents at least 8 unique species, confirmed via average nucleotide identity (ANI) (Table S3) and digital DNA-DNA hybridization (dDDH) (Table S4) (18). This genome set represents strains isolated from diverse marine environments/niches, including coastal biofilms, hydrothermal vents, and algal-associated isolates (Table S5). Using these thresholds, 11 *pontiacus*, 5 *profundi*, 3 *faviae*, and 2 *dubius* strains were identified. One unclassified strain, DSM 110093, clustered closely with the two *S*. *dubius* strains (ANI = 0.92) and carries a plasmid that appears conserved in members of the species. Consequently, DSM 110093 was assigned to the *S. dubius* classification for subsequent analyses. Additionally, some discrepancies existed between the ANI and dDDH-deduced species designations. Strains M368, SC1-11, M342, and SC7-37 clustered together as a single species according to dDDH scores (>78.7) but form two separate clusters (M342/SC7-37 and M368/SC1-11) by ANI scores (Tables S3 and S4). Since these strains share common features, including a highly conserved flagellar plasmid, they were collectively grouped under the *S. mediterraneus* species for this analysis. However, sub-clustering observed in the core genome tree, which is congruent with ANI scores, suggests that further subspecies-level differentiation may be warranted (Fig. S2). Certain species clustered closely based on both ANI and dDDH scores, indicating a possible recent common ancestor. For example*,* all strains designated as *S. profundi, faviae, and dubius* shared ANI scores >0.88, a finding corroborated by the core genome tree. Conversely, ANI scores for other strains (e.g., S190, S223, SK011, JK07, and SK012) were <0.8 when compared to all other genomes, suggesting each strain represents a distinct species within the genus.

**Fig 1:**
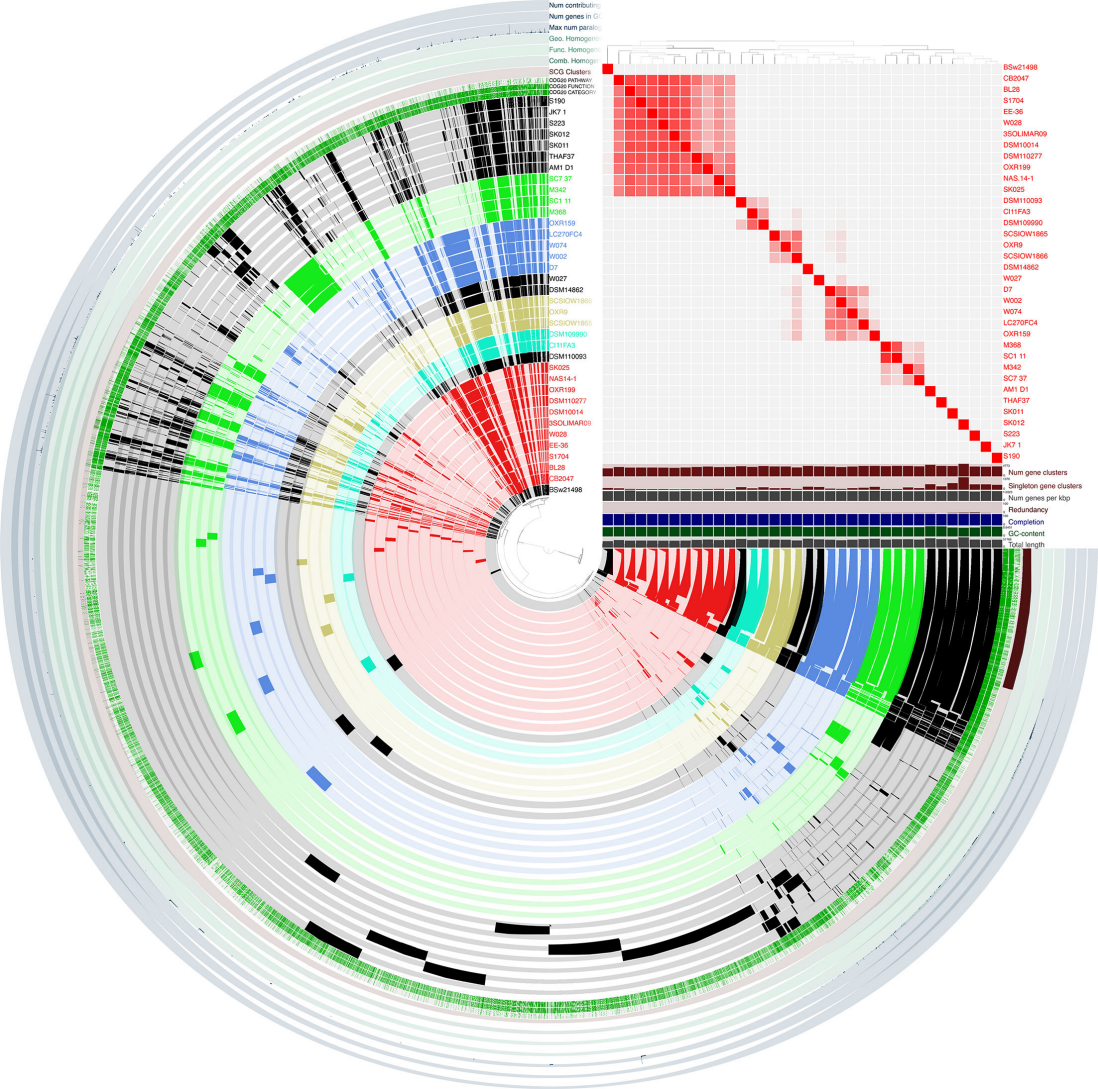
Pangenome analysis of the 36 closed *Sulfitobacter* genomes in the analyzed set. The circular diagram represents the core and accessory genome composition, with species containing multiple representatives color-coded as follows: red*—S. pontiacus*, teal*—S. dubius*, brown*—S. faviae*, blue*—S. profundi*, green*—S. mediterraneus*. Other species, including strains that have not yet been taxonomically classified, are indicated in black. The heatmap inset in the upper right corner displays ANI comparisons. The graphic was generated using Anvi’o v8 and pyANI for genome clustering.

Analysis of the 36 closed *Sulfitobacter* genomes reveals substantial genomic diversity in both chromosome size (2.91–4.78 Mb, median 3.32 Mb) and plasmid content. *S. pontiacus* strains have the smallest chromosomes (mean ~3.0 Mb), whereas *S. mediterraneus* strains tend to carry larger chromosomes (mean ~3.8 Mb). Genomes are rich with extra chromosomal replicons, with each strain harboring more than three plasmids, on average, and plasmid-encoded sequences comprising ~15% of the total genome (Table S6). The proportion of plasmid-encoded DNA varies by species, accounting for nearly 18% of *S. pontiacus* genomes but only ~8% of *S. mediterraneus* genomes. *S. faviae* DSM 14862 shows the highest proportion, with 28.3% of the genome being plasmid-encoded. Core genome analysis identified 98 genes conserved across all sequences using a 90% presence threshold across genomes (i.e., ≥90% amino acid identity in ≥90% of genomes), primarily mapping to essential cellular processes, such as amino acid metabolism, energy production, and translation (Table S7). All core genes are chromosomally encoded across all strains. Pangenome construction revealed 19,236 total genes, highlighting extensive genetic diversity beyond the conserved core (Fig. 1). A core genome-based phylogenetic tree (Fig. S2) facilitated identification of additional conserved genes across subsets of species. For example, 398 genes are conserved between *S. pontiacus* and *litoralis,* but not other species. Of these, 229 genes are functionally annotated (Table S8) and include signal transduction mechanisms, cell wall/membrane/envelope biogenesis, and coenzyme transport and metabolism. Similarly, sister species (e.g., *S. dubius*, *faviae*, and *profundi*) harbor homologous, yet distinct, genes relative to other *Sulfitobacter* species. This includes genes associated with signal transduction mechanisms, amino acid transport and metabolism, and cell motility. Notably, these species encode on their chromosomes structurally distinct flagellar genes relative to *S. pontiacus* and *S. mediterraneus* strains, which are plasmid borne. Analysis of the pangenome also revealed differences in gene sequences for important metabolic pathways, including thiamine and cobalamin synthesis.

Ribosomal RNA (*rrn*) operon copy number varies systematically by species: *S. mediterraneus* (two copies), *S. pontiacus* (three copies)*,* and *S. dubius* strain DSM109990 (four copies). Notably, *rrn* operon localization differs between species, with *S. pontiacus* and *S. dubius* DSM109990 possessing plasmid-borne *rrn* operons, while *S. mediterraneus* strains maintain all *rrn* operons on chromosomes. Additionally, *S. profundi* strains possess four *rrn* operons, two of which appear “coupled” with other mobile elements, specifically integrases and transposases, suggesting recent genome transposition. Finally, in *S. profundi* D7, one *rrn* cluster appears disrupted, as a hypothetical protein and a putative glycosyl transferase gene are found within the 23S rRNA gene (Fig. S3). No conserved boundary signatures or detectable homology consistent with the described IStron families were observed.

Chromosomes encoded a consistent tRNA complement, ranging from 42 to 52 predicted tRNAs per chromosome across strains (Table S9). A minority of plasmids (~10%) carried two to three predicted tRNAs. Plasmid-borne tRNAs were restricted to *rrn* operon-harboring plasmid replicons (and co-localized with these ribosomal RNA genes) rather than being widespread across plasmid sets. Additionally, CRISPR-Cas loci were not detected on any of the *Sulfitobacter* replicons, nor were any robust CRISPR array predictions consistent with canonical repeat spacer architectures detected.

### Plasmid conservation and network analysis

**Fig 2:**
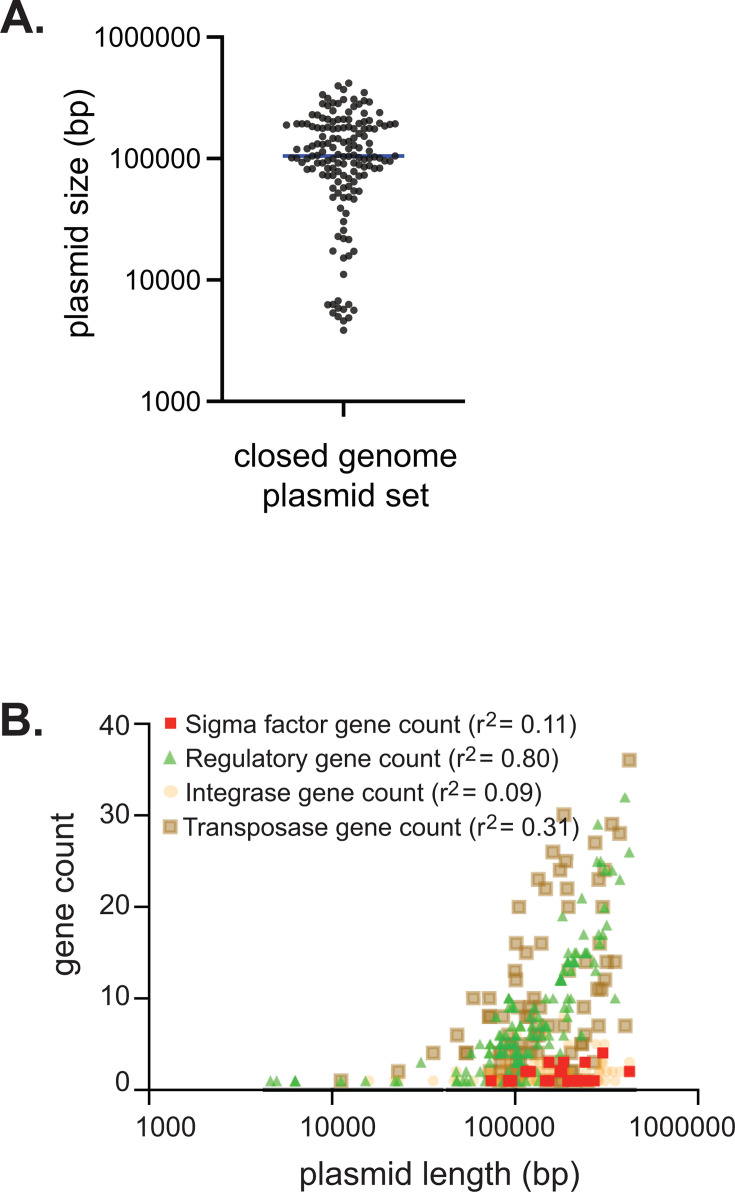
Structural and functional diversity of *Sulfitobacter* plasmids. (**A**) Distribution of plasmid sizes across the closed genome set showing a bimodal size distribution. (**B**) Relationship between plasmid size and gene content for selected functional categories. R-square values (Pearson correlation) for each gene category are provided in parentheses.

Across the *Sulfitobacter* genomes included in this study, plasmids show variation in length (3.8–417 kb, median 105.6 kb). The distribution of plasmid sizes reveals a bimodal pattern, with most plasmids clustering around 5–150kb, and a smaller population of very large plasmids (>200 kb) (Fig. 2A). This size distribution reflects functional specialization, with larger plasmids typically harboring essential lifestyle functions, such as flagellar motility systems, while smaller plasmids often encode accessory metabolic functions or regulatory elements (Table S10).

**Fig 3:**
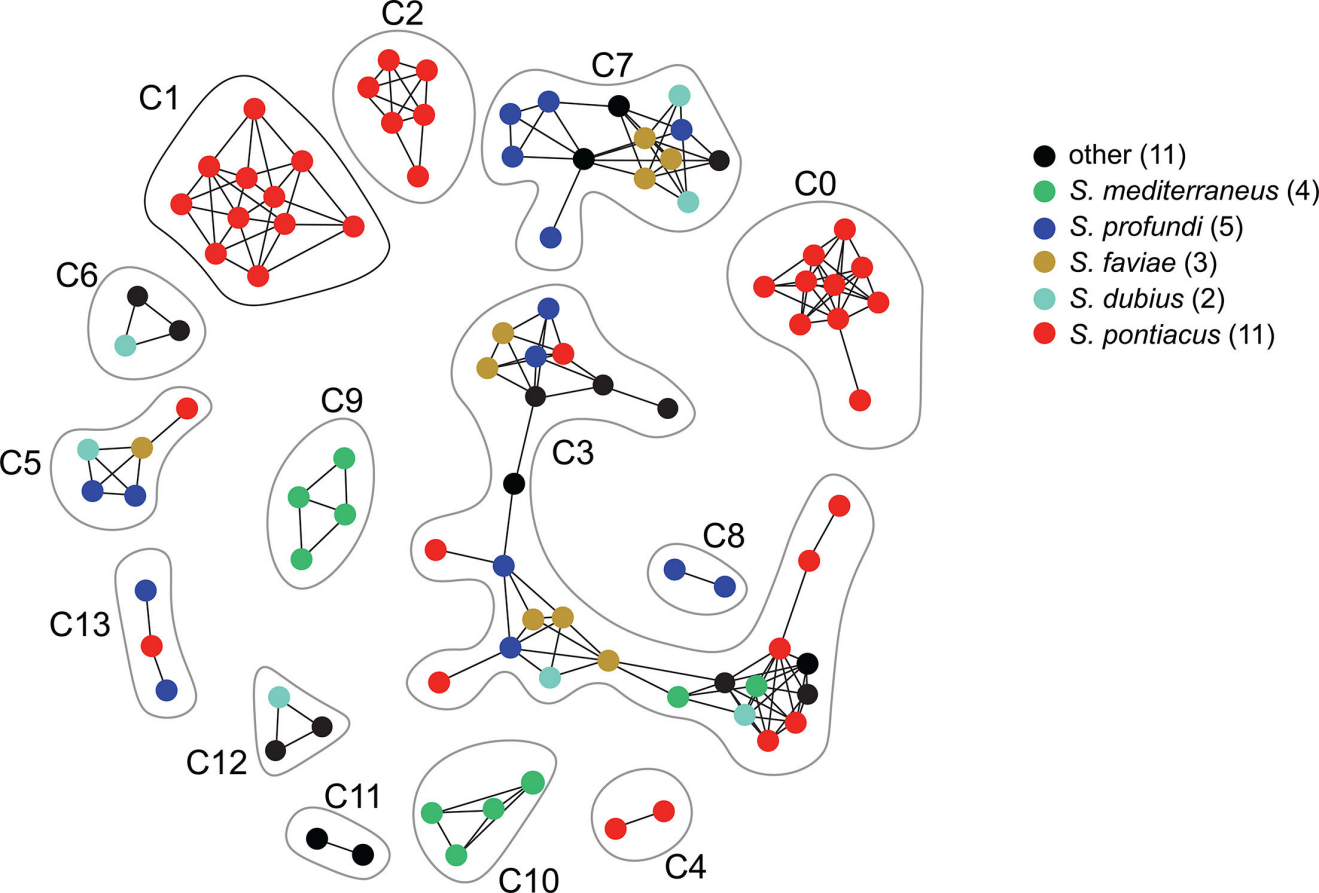
Network analysis of the *Sulfitobacter* plasmid data set yielded 14 communities. Nodes represent individual plasmids, with edges indicating significant sequence similarity (containment distance ≤ 0.5) and structural relatedness (DCJ-Indel distance ≤ 4). Each node is color-coded to reflect species designation for species with at least two representative strains, following the color schematic in Fig. 1 and represented by the key here, where the number in parentheses indicates the number of strains. Plasmids not in communities (i.e., singletons) are not shown in this image. The network was generated using pling, with default parameters. Detailed information on community composition is available in Table S11.

Plasmid abundance varies significantly between strains, with 33 of 36 strains (92%) carrying three or more plasmids and 10 strains harboring six or more plasmids (average 4.25 plasmids/strain). Three strains (SK011, BSw21498, M368) possess fewer than three plasmids. Network analysis of the 153 plasmids in the 36 genome set identified 14 primary “communities” (i.e., clusters of plasmids that share significant sequence similarity), designated C0-C13, containing 97 plasmids (63% of total), with remaining plasmids forming singletons (Fig. 3). Communities varied dramatically in terms of genetic diversity represented. Some show remarkable homogeneity, with plasmids exhibiting >95% nucleotide identity, while others contained highly diverse plasmids grouped only by shared functional modules. Species-specific conservation patterns were striking. *S. pontiacus* plasmids formed three distinct clusters: (i) C0 contains 10 large plasmids (200–395 kb); (ii) C2 contains six smaller homologous plasmids (54–72 kb); and (iii) C1 contains 11 large (~176 kb) plasmids encoding the sole flagellar gene cluster for each strain. These flagellar plasmids form a single, highly conserved cluster with >95% nucleotide identity and high synteny across. Similarly, the four *S*. *mediterraneus* flagellar plasmids, which are about half the size (~92 kb) of the *S. pontiacus* flagellar plasmids, also show exceptional conservation within the species (C9). In contrast, several communities contain plasmids from multiple *Sulfitobacter* species (e.g., C5 and C7 contain between 5 and 13 plasmids from 4 or more different species). These communities are noteworthy as plasmids in C7 tend to further subdivide into subcommunities (Table S11) by species, while C5 consists of small plasmids (<10 kb), a rarity among *Sulfitobacter* genome sequences (Table S6). Additionally, non-identical plasmids from different isolates encode gene communities with high sequence conservation (Table S12). While identical sequence lengths vary (~500 to ~26,000 bp), this finding highlights the widespread conservation of specific gene clusters and genus-wide genome mosaicism.

Mosaic fragments were most evident in large plasmid communities with further division into subcommunities (e.g., C0, C3, C7, C11–13; Tables S11 and S12). Each community consists of multiple plasmids that exhibit varying degrees of homology, with subcommunities representing more closely related plasmids within a given community. For example, 28 plasmids were distributed in C3, which has 21 subcommunities. Twenty of those plasmids also carried mosaic fragments. In addition, 15 plasmids in C3 show mosaicism and encode multiple transposases. Replicon typing revealed distinct patterns in plasmid clustering, with certain Rep-types preferentially distributed among specific communities and subcommunities. For instance, RepB was the most frequently observed type and was predominantly associated with plasmids in C0 (Fig. 3). RepA and DnaA-like replication proteins were also identified across multiple subcommunities, where they showed high sequence identity at the protein level, suggesting functional conservation across diverse plasmids (Table S10).

The above analyses also provide evidence for apparent plasmid loss in some genome assemblies. Notably, strain BSw21498 genome sequence lacks the highly conserved flagellar plasmid found in other sequenced *S. litoralis* representatives, the genus to which this strain is expected to belong based on whole-genome ANI. Consistent with this, the flagellar gene cluster is also absent from the BSw21498 chromosome. Additionally, the closed *S. dubius* CI.11.F.A3 genome is missing a *rrn* operon-containing plasmid present in the other *S. dubius* strain, DSM10990. However, both strains retain chromosomal *rrn* operons.

### Functional content of conserved plasmids

Flagellar gene clusters represent the most conserved plasmid-borne functions in *Sulfitobacter* species. All *S. pontiacus* strains possess an ~175 kb flagellar plasmid encoding ~45 flagellar biosynthesis genes, with exceptional sequence conservation (>95% nucleotide identity) and synteny. These plasmids also encode the flagellar-specific σ^28^ regulatory element, as well as an extracytoplasmic function (ECF) σ^70^ family sigma factor. ECF σ-factors are a subclass of σ^70^-family factors involved in responding to environmental stress, particularly in controlling genes related to cell surface functions (19, 20).

Analysis of σ-factor gene distribution across all plasmids revealed that 33 plasmids (~20% of total) encode at least one putative σ-factor, primarily within the σ^70^, σ^28^, and σ^54^ families. Three plasmids harbored by *S. dubius* strain CI.11.F.A3, *S. faviae* strain SCSIO W_1865, and *S. profundi* strain LC.270.F.C4 encode three unique σ^70^ family RNA polymerase σ-factors, which are likely ECF σ-factors based on predicted functional domains (two rather than four). All *S. mediterraneus* strains also harbor a conserved 92 kb flagellar plasmid. Comparison between *S. pontiacus* and S. *mediterraneus* flagellar plasmids indicates gene synteny with 80% query cover and 70% sequence identity over the 40 kb flagellar gene region, suggesting shared evolutionary origin despite species-specific adaptations (Fig. S4). Finally, another plasmid showing conservation across the seven completed *S. pontiacus* genomes appears to encode critical biofilm-associated functions. In most of the strains, the plasmid is ~55 kb in length, but extends to 72 kb in two strains (CB2047 and S1704). This plasmid, denoted pSpoCB-4 in strain CB2047, encodes several membrane-associated genes (e.g., glycosyltransferases) and the biofilm-associated gene cluster *rfbABCD*, which is predicted to encode proteins involved in polysaccharide biosynthesis, critical for biofilm formation (8).

### Mobility and transfer systems

Classification of plasmid mobility revealed distinct size-function relationships. Of the 153 *Sulfitobacter* plasmid data set, 36 (24%) are predicted to be conjugative, encoding complete T4SS machinery, with an average length of 143 kb. Fourteen (9%) are predicted to be mobilizable, harboring *mobQ* family relaxases and averaging only 20 kb in length (Table S13). The majority (~67%) of the plasmid data sets are designated as non-mobilizable, given they lack both *mobQ* and T4SS genetic elements. This plasmid class averages 236 kb in length, and ~8% of these putative non-mobile plasmids encode type II secretion systems, suggesting they play roles in host interactions (Table S10). Plasmid replicons were additionally evaluated for phage-plasmid signatures, including hallmark phage genes and phage-like modular architectures. Screening with geNomad identified no plasmid sequences classified as either viral or integrated prophage. Consistent with this result, manual inspection of plasmid annotations did not reveal co-localization of phage structural or packaging modules, such as terminase, portal, capsid, or tail genes, that would support a phage-plasmid interpretation. Plasmid mobility/recombination proteins were further categorized using protein domain-based evidence. The majority (92%) of these 942 plasmid-encoded elements were classified as DDE-family transposases (77%), tyrosine recombinases (8%), and serine recombinases (7%); HUH-family transposases were not evident in the plasmid data set (Table S14). Mobilization proteins (e.g., relaxase/Mob) were evident in only 13 plasmids, all of which fell on the lower end of the size spectrum at 4,986–48,252 bp (Table S13).

### Transposable and insertion sequence element dynamics

Transposase abundance varies widely among plasmids: 10% contained ≤20 transposases (Table S10), with no correlation to GC content, replication type, or T4SS presence. Most σ-factor encoding plasmids (17 of 33) lack transposases entirely, while 4 plasmids carry only one; 82% also lack annotated integrases. Conversely, 63% (76/120) of non-σ-factor plasmids encode transposable elements. Notably, when considering the full *Sulfitobacter* genome set (chromosomes plus plasmids), transposases were on average threefold more abundant than σ-factors, yet variation among genomes was substantial, and no significant correlation was observed between total transposase copy number and sigma factor count (R^2^ = 0.27; Table S15). Despite this pattern, some σ-factors appear to have been mobilized by transposable elements: pDSM110277_a (239 kb) carries two σ-factors and one anti-σ-factor (Fig. S5). These σ-factors are in proximity to one another and are flanked on either side by tn3- and *mu-*type transposases.

**Fig 4:**
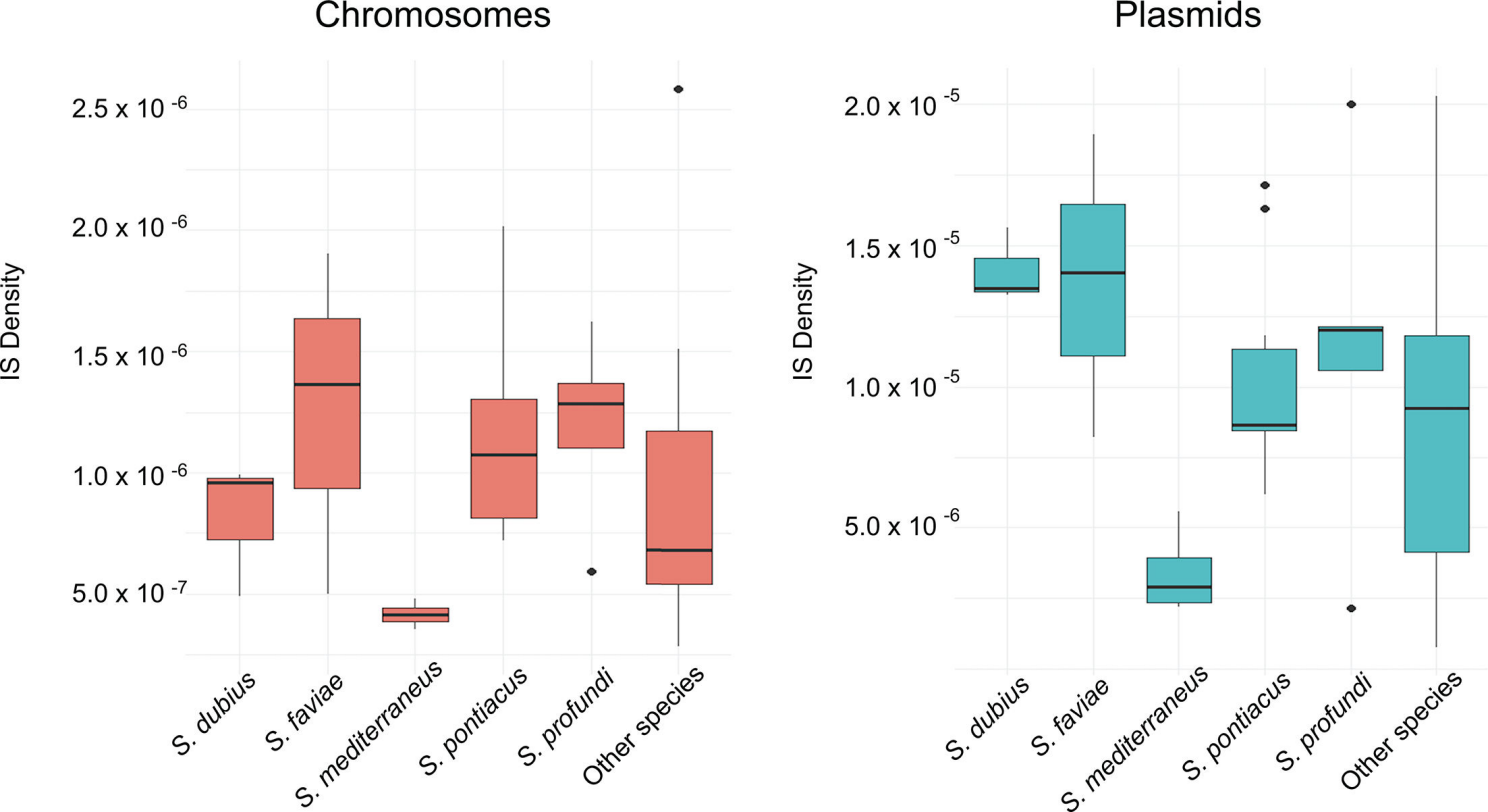
IS density across replicon type in the *Sulfitobacter* closed genome set. IS densities on chromosomes (salmon) and plasmids (teal) are indicated for the five most represented species, with remaining strains grouped as “other species.” Each box represents the interquartile range; points indicate outliers. Plasmid IS density is significantly higher than chromosome IS density across the data set (Wilcoxon rank-sum test, *P* < 0.001).

Examination of gene content relationships across all plasmids reveals distinct functional trade-offs (Fig. 2B). Regulatory gene content shows the strongest positive correlation with plasmid length (r2 = 0.80), suggesting that larger plasmids serve as platforms for complex regulatory networks. In contrast, transposase gene count shows only moderate correlation with plasmid size (r2 = 0.31), while σ-factor and integrase gene counts show weak correlations with length (r2 = 0.11 and 0.09, respectively). Insertion sequences (IS) are also widespread and display distinct patterns of distribution across chromosomes and plasmids. IS elements are significantly more concentrated in plasmids than chromosomes (paired Wilcoxon signed-rank test, *P* < 0.001) (Fig. 4). Species-specific patterns emerged in both IS density and family distribution. *S. mediterraneus* strains showed lower IS densities in both chromosomes (4.16 × 10⁻⁷) and plasmids (3.39 × 10⁻⁶) compared to other *Sulfitobacter* chromosomes (1.13–1.26 × 10⁻⁶) and plasmids (1.03–1.37 × 10⁻⁵). IS family distribution also reveals species-specificity: *S. pontiacus*, *S. faviae*, and *S. mediterraneus* chromosomes are dominated by IS3 elements, while *S. profundi* chromosomes are enriched with IS256 elements. Plasmids also showed distinct preferences, with *S. pontiacus*, *S. profundi*, *S. faviae*, and *S. mediterraneus* plasmids appearing enriched for IS66, IS30, IS21, and IS5 family elements, respectively.

Amongst the *Sulfitobacter* plasmid data set, the IS3, IS6, and IS256 family transposases are highly recurrent, suggestive of active transposable elements and horizontal gene transfer within the plasmids harboring them. For example, strain 3SOLIMAR09 possesses a unique nitrous oxide reductase *nosZDFY*L regulon flanked by transposases and integrases on plasmid p1, showing high homology to *Phaeobacter* (*Roseobacteraceae*) and *Paracoccus* (*Paracoccaceae*) strains rather than other *Sulfitobacter* representatives. Another intriguing integration was observed in NAS-14.1 plasmid 1 (P1). Although all *S. pontiacus* strains encode a thiosulfate oxidation gene cluster (*sox*), NAS-14.1 encodes an additional *sox* cluster located on plasmid P1. This cluster is distinct from the chromosomal *sox* cluster (70% nucleotide identity), suggesting NAS-14.1 may have different sulfur transformation efficiencies compared to related strains.

The functional genes most often associated with transposases and IS elements are those likely to confer fitness advantages in the face of environmental pressures, including multicopper oxidase domain-containing proteins and cation diffusion facilitator family transporters, expected to mediate resistance to metals (21). Furthermore, genes encoding putative transketolases, FAD-dependent oxidoreductases, and various dehydrogenases suggest these plasmids are involved in key metabolic pathways, potentially associated with specific ecological niches or competition with other microorganisms. In addition, the data set included several transcriptional regulators, such as LysR and AraC family transcriptional regulators, suggesting plasmid presence may influence the expression of not only plasmid-encoded genes, but possibly of the remaining host genome. The presence of these plasmid-localized transcriptional regulators suggests plasmids in *Roseobacteraceae* members may be active participants in bacterial adaptation and survival strategies (12).

### Chromosomal integration of a plasmid in *S. pontiacus* strain CB2047

*S. pontiacus* strain CB2047 exhibits a unique genomic architecture where a large plasmid homologous to 200–375 kb plasmids in all other closed *S. pontiacus* genomes appears integrated into the chromosome at an *rrn-*associated locus. This 280 kb integrated element encodes typical plasmid-associated features, including two type II toxin-antitoxin systems (IV89_000201-IV89_000202, and IV89_RS00057-IV89_RS00058) and a plasmid partitioning system (ParAB-like elements). Integration was confirmed through long-read mapping using Nanopore sequencing data, with multiple Nanopore reads spanning both chromosome-element junctions (Tables S16 and S17).

The integration results in a configuration where the integrated plasmid is flanked by two highly similar *rrn* operons (~5.6 kb, differing by only two nucleotides) while retaining an intact plasmid-encoded operon within the integrated element. This contrasts with all other *S. pontiacus* strains, which have three *rrn* operons distributed as two chromosomal and one plasmid-borne copies, whereas CB2047 contains all three *rrn* operons chromosomally. Inspection of the junction regions revealed no evidence of mosaic or truncated rrn operons; instead, all *rrn* copies remain full-length. The integrated plasmid region in CB2047 is 59.2% GC, compared with 60.5% GC for the chromosome, and falls within the GC range observed for the conserved *S. pontiacus* plasmid lineage (plasmid community C0) (59.0%–59.5% GC), consistent with integration of a lineage-typical plasmid rather than acquisition of a compositionally distinct element.

## DISCUSSION

The influence of mobile genetic elements on the evolutionary features of specific *Roseobacteraceae* members has been documented and tied to genome evolution, adaptation, and trait acquisition (4, 22). Our comprehensive analysis of 153 plasmids across 36 complete *Sulfitobacter* genomes provides further evidence that plasmids function as primary drivers of evolutionary diversification and ecological adaptation within this marine genus. The extensive conservation of species-specific plasmids, combined with evidence for inter-species transfer and integration events, reveals a complex evolutionary landscape where plasmids serve dual roles: as repositories of conserved adaptive traits and platforms for genetic innovation. Importantly, our conclusions regarding gene localization and replicon architecture rely exclusively on closed genomes. In plasmid-rich lineages, such as *Sulfitobacter*, draft assemblies can misassign contigs, collapse repetitive regions, or fail to resolve integration events, leading to incorrect designation of genes as plasmid- or chromosome-borne. Restricting replication-level inferences to complete genomes, therefore, increases confidence in the identification of *rrn* operon placement, IS enrichment patterns, and the chromosomal integration event observed in CB2047.

The remarkable conservation of flagellar plasmids within *S. pontiacus* (>95% identity across 11 strains) and *S. mediterraneus* (>88% identity across 4 strains) species contrasts sharply with the diversity observed in cross-species comparisons. This is suggestive of strong selective pressure for maintenance of motility functions within specific ecological niches while allowing species divergence. This pattern mirrors findings in other *Roseobacteraceae,* where lifestyle-determining genes cluster on plasmids, enabling rapid phenotype switching through plasmid gain or loss (7, 8).

The 8.8-fold enrichment of IS elements in plasmids compared to chromosomes provides quantitative evidence for plasmids serving as evolutionary hotspots in this genus. This finding is consistent with evidence from global bacterial genome databases (23). Moreover, the introduction of IS into plasmids has been linked to plasmid host range (24). Thus, this observed enrichment of IS elements in *Sulfitobacter* plasmids, combined with extensive mosaicism evident across the majority of the plasmid data set, is suggestive of active genetic exchange mechanisms that facilitate rapid adaptation.

The presence of σ-factors on 22% of plasmids, particularly ECF-type σ-factors associated with stress response, suggests *Sulfitobacter* plasmids actively influence global gene regulation beyond their encoded functions. This finding supports previous observations in *Dinoroseobacter shibae*, where plasmid-encoded σ-factors were suggested to modulate chromosomal gene expression (11), indicating a regulatory network spanning episomal and chromosomal compartments. However, this hypothesis has not yet been experimentally validated. Additionally, the negative correlation between σ-factor presence and transposase abundance is suggestive of functional constraints on mobile element activity in regulatory plasmids, potentially preserving essential regulatory circuits while allowing flexibility in accessory functions.

The functional clustering analysis provides quantitative support for a plasmid specialization model in *Sulfitobacter*. The clear separation between regulatory-rich and mobility-rich (i.e., integrases and transposases) plasmids suggests that evolutionary pressures favor functional compartmentalization, where essential adaptive functions are maintained on stable, regulatory-rich platforms while genetic innovation occurs on highly dynamic, transposon-rich replicons. Where obvious, this partitioning of functionality is aligned with specific replication modules. DnaA-like containing plasmids are enriched in regulatory genes relative to the RepABC containing plasmids, for which integrases and transposases show a bias. RepABC has been characterized as a self-contained replication and partitioning system that is common in Alphaproteobacteria (25). Conversely, DnaA-like Roseobacteraceae plasmids may depend upon, or work cooperatively with, host-derived replicon machinery (26). This specialization likely reflects the dual evolutionary strategy observed in marine copiotrophic bacteria, where core adaptive capabilities must remain stable while accessory functions evolve rapidly in response to environmental fluctuations (27).

The plasmid localized IS element enrichment varies across species, most notably with *S. pontiacus* and *S. mediterraneus* showing distinct distributions that may reflect ecological adaptations. These findings suggest *S. pontiacus* may occupy more dynamic environments requiring greater genomic plasticity, while *S. mediterraneus* may inhabit more stable niches with reduced selective pressure for IS-mediated genome modifications, a question that could be addressed with a more robust closed genome set. IS elements may drive this proposed plasticity by disrupting or regulating genes, facilitating genome rearrangements (e.g., inversions, deletions, and duplications), and mediating horizontal transfer of adaptive genes (28–31). Families, such as IS21 and IS110, actively mobilize plasmid genes, which can lead to the formation of novel genes, supporting metabolic diversification (32). However, high IS densities create a trade-off between adaptability and genomic stability (33), which may explain the aforementioned observed regulatory plasmid constraints.

The chromosomal integration event observed in *S. pontiacus* CB2047 likely represents a significant evolutionary innovation. Integration at the *rrn-*associated operons, most parsimoniously explained by homologous recombination involving highly similar rrn operons, demonstrates how *Roseobacteraceae* mobile elements may transition from episomal to chromosomal inheritance. The extremely high sequence identity between flanking *rrn* operons suggests that recombination, followed by gene conversion, could produce the observed seamless configuration without detectable hybrid boundaries. This high degree of sequence similarity would enable RecA-mediated recombination, without requiring specialized integration systems.

While we considered alternative mechanisms, such as site-specific integration characteristic of integrative conjugative elements, we found no evidence for hallmark features, including integrase genes at element boundaries or flanking repeat sequences, indicating *att* site recombination. The conservation of this element as an extrachromosomal plasmid in closely related strains (Fig. S6, Table S18) further supports homologous recombination as the integration mechanism. This interpretation is consistent with reports of chromosome-chromid fusion via homologous recombination between rRNA operons in *Cupriavidus necator*, which similarly preserved intact rRNA operons without detectable hybrid boundaries (34). Such integration events potentially stabilize beneficial traits while maintaining plasmid-specific regulatory and partitioning machinery. This proposed mechanism further parallels integration events documented in other bacterial systems, where similarly large plasmids (>100 kb), such as the F plasmid in *Escherichia coli* (35), and toxin-encoding plasmids in *Clostridium* species (36), integrate into their hosts’ genomes. The near identity of the *S. pontiacus rrn* operons also suggests that recombination between these loci could occur readily and bidirectionally, potentially enabling both integration and excision events.

Collectively, this analysis highlights that integration of ANI, dDDH, and plasmid content analysis provides a more robust framework for species delineation than traditional approaches, particularly for groups with complex evolutionary histories shaped by horizontal gene transfer, as is evident in *Roseobacteraceae* (3). This work supports recent taxonomic revisions within the *Sulfitobacter* genus and identifies additional strains ripe for systematic evaluation. The clear separation of *S. algicola* (now *Parasulfitobacter* [37]) and the identification of potentially misclassified strains, such as JL08, demonstrate how plasmid content analysis can inform taxonomic decisions. However, apparent plasmid loss events in some genome assemblies (e.g., missing flagellar plasmid in BSw21498) emphasize the challenges of culture-based genomics and potential assembly artifacts. Nonetheless, future taxonomic work should consider plasmid conservation patterns alongside standard genomic metrics.

The patterns observed in *Sulfitobacter* plasmids likely reflect broader evolutionary strategies of copiotrophic bacteria in marine niches, where fluctuating environmental conditions favor genetic flexibility. The maintenance of large, conserved plasmids encoding essential functions (e.g., flagellar motility, biofilm formation) alongside smaller, more genetically variable elements suggests a tiered adaptation strategy where core adaptive functions remain stable while accessory capabilities evolve rapidly.

## MATERIALS AND METHODS

### Genome selection and quality assessment

We first analyzed 374 *Sulfitobacter* genomes consisting of 27 characterized species (Table S1). Genomes were screened for completeness (>95%) and contamination using CheckM (Table S2). ANI was calculated between all genome pairs to identify highly similar strains. For strains exhibiting high ANI similarity (>99.9%), a single representative strain was selected for inclusion in subsequent analyses. The resulting dereplicated genome set contained 129 genomes representing 22 species. It should be noted that *S. algicola* has been recently proposed for taxonomic reclassification to a new genus (*Parasulfitobacter*) (37), which is supported by our analysis, but not yet evident in the NCBI Taxonomic classification scheme (Fig. S1). Consequently, the single *S. algicola* genome in the dereplicated set was excluded from subsequent analyses to maintain consistency in genus-level comparisons.

For detailed plasmid analysis, we focused exclusively on closed genomes to ensure complete plasmid sequence confirmation. This data set consisted of 32 publicly available *Sulfitobacter* genomes plus four additional genome sequences closed during this study (EE-36, NAS-14.1, 3SOLIMAR09, DSM 10014), yielding 36 total closed genomes representing 8 unique species. These four additional strains represent some of the earliest isolated, sequenced, and studied *Sulfitobacter* isolates (38). Collectively, the 36 genomes represent strains isolated from diverse marine environments/niches, including coastal biofilms, hydrothermal vents, and algal-associated isolates (Table S5). Species boundaries were confirmed using ANI (Table S3) and dDDH (Table S5) with established thresholds of 95%–96% ANI and 70% dDDH for species delineation (27).

### Genome resequencing and closure of additional *Sulfitobacter pontiacus* strains

Strains EE-36, NAS-14.1, 3SOLIMAR09, and DSM 10014 were routinely cultured at 25°C in the dark at 200 rpm on Standard Marine Media, an artificial seawater medium supplemented with 0.11% yeast extract and 0.2% tryptone as previously described (39). DNA extraction was performed using a Qiagen DNeasy kit according to the manufacturer’s protocol. DNA yield was determined using a Nanodrop UV-Vis Spectrophotometer. The extracted DNAs were sequenced at the Microbial Genome Sequencing Center (Pittsburgh, PA, USA). For short-read sequencing, the Illumina NextSeq 2000 platform (2 × 150 bp paired-end) was used. The genomes of four *S*. *pontiacus* strains (EE-36, 3SOLIMAR09, NAS-14.1, and DSM 10014) were closed using the Oxford Nanopore Technologies platform. Quality control, adapter trimming, and quality filtering of the short reads were performed using fastp (40). Quality filtering of the long reads was performed in filtlong. Genomes were assembled using Unicycler (normal mode, v0.49b) by combining the short reads from Illumina sequencing and long reads from Nanopore sequencing (41). Assembled genomes were visualized using Bandage software (42). Annotations were performed using Prokka (43). Genome sequences were deposited in NCBI (SAMN42123194, SAMN42123195, SAMN42123196, SAMN42123197).

### Phylogenetic and pangenome analysis

Core genome phylogenetic trees were constructed using single-copy core genes identified across all 36 closed genomes. The pangenome was built in Roary (44). A total of 124 genes were determined as core genome (90% homology cut-off), and a single copy of these genes was extracted from each of these sequences. The amino acid sequences were aligned using MAFFT with default parameters (45). A maximum-likelihood core-genome phylogenetic tree using RAxML was used to construct a phylogenetic tree from the final alignment with the PROTGAMMAAUTO option for automatic model selection. The robustness of branching was estimated with 100 bootstrap replicates (46).

An alternative pangenome was built using Anvio (version 8) (47). Functional annotations of proteins were determined according to Clusters of Orthologous Groups using Anvio. Pangenomes were constructed in three different ways: using whole genomes, chromosomes only, and plasmids only. Genome similarity was calculated using the PyANI program with default parameters in Anvi’o (48). Homology of plasmids and chromosomes was assessed with BLASTn, and alignments were made with progressive Mauve (49).

### Plasmid characterization and network analysis

Plasmids were identified from closed genome assemblies and analyzed using pling (28) to calculate genetic distances. Network analysis was performed with clustering based on sequence similarity thresholds, generating primary clusters that were further subdivided into subclusters based on genetic distance. Plasmids sharing >40% query coverage and >60% nucleotide identity were considered related.

Plasmid mobility was classified based on the presence of transfer machinery: conjugative plasmids encoded complete type IV secretion systems (T4SS), mobilizable plasmids carried *mobQ* family relaxases without T4SS, and non-mobilizable plasmids lacked both systems. Replicon typing was performed to identify plasmid replication systems (RepA, RepB, RepABC, DnaA-domain containing, RepO, RepY, RepW, RepL, RepC-soli) (26, 27).

### Other bioinformatic analysis

The insertion elements were predicted using ISeScan (50). To quantify IS density, we calculated the number of predicted IS elements per base pair for each replicon (chromosome and plasmid) within every genome. IS density statistics were then summarized at both the strain and species levels, and Wilcoxon signed-rank tests were performed to assess differences between chromosomes and plasmids. Relaxase (MOB) families, predicted mobility, and replicon typing were inferred using MOB-suite (51). Each plasmid FASTA was analyzed independently. Identification and analysis of plasmid mosaicism were done with a custom Python script as described by Pesesky et al. (52). The script employed the BLASTn tool to find regions of high sequence similarity, specifically looking for segments that matched the criteria for mosaicism: at least 500 bp with 100% identity between two plasmids that otherwise share less than 93.9% overall sequence identity. After running BLASTn, the script filtered out results to those meeting these criteria. Later, annotations of the mosaic regions were determined. Plasmid clustering and analysis were done using pling, which calculates rearrangement distances between plasmids to identify and cluster related plasmid sequences (53). We utilized the default settings of “pling” with the align option. Following the pling output, we developed a custom R script to generate visual representations of the clusters. To determine the replication initiator protein (Rep-type) of *Sulfitobacter* plasmids, a local BLASTp search was performed. A custom reference database was constructed using known Rep protein sequences from *Roseobacteraceae* and related Alphaproteobacteria. All predicted protein-coding sequences from each plasmid were extracted using Prodigal annotations from GenBank files, and BLASTp was run. The best hit for each plasmid was selected based on percent identity, e-value, and alignment length. Rep-types were annotated according to the closest matching protein in the reference database, and results were compiled into a supplementary table for comparison with plasmid clustering and network data. tRNA genes were predicted using tRNAscan-SE 2.0 bacterial mode (54). CRISPR-Cas systems were screened by PADLOC (55) and CRISPRCasTyper (56) with default settings. All plasmid sequences were screened using geNomad, with default parameters, to assess the presence of phage-plasmid signatures (57). The geNomad end-to-end workflow was run on each plasmid FASTA, and per-sequence classifications were used to identify any plasmid sequences assigned to viral or integrated-prophage categories. Exhibit ambiguity in “integrase” and “transposase” labels derived from automated annotations all plasmid mobility/recombination proteins were analyzed using Pfam HMMs with HMMER (58) and assigned to mechanistic classes based on diagnostic domains, including DDE-family transposases, tyrosine recombinases, and serine recombinases; mobilization proteins (e.g., relaxase/Mob) were tracked separately. HUH-family transposases were also queried but were not prevalent in the plasmid data set (Table S17). To assess whether the *rrn-*disrupting IS insertion might represent an IStron or other self-splicing intron-associated element, we extracted the insertion junction and flanking sequence and screened it for canonical group I/II intron boundary motifs and for sequence homology to reported IStron-associated intron ends using nucleotide similarity searches (59).

### *S. pontiacus* CB2047 plasmid integration event validation

The chromosomal integration event in *S. pontiacus* CB2047 was confirmed through long-read Nanopore sequencing data mapping. To confirm the integration of a plasmid into the chromosome of *Sulfitobacter pontiacus* strain CB2047, long-read sequencing data generated using Oxford Nanopore Technology were analyzed. We designed a 15 kb artificial junction reference sequence spanning the putative integration site between plasmid and chromosomal regions. Using Minimap2 (60), we aligned raw reads to these junction sequences. Multiple long nanopore reads (up to 80 kb in length) mapped contiguously across the entire artificial junction region, with no breaks or drops in coverage. These reads aligned across both flanks of the junction (from chromosomal to plasmid regions). High mapping quality scores and low divergence values confirmed the accuracy of these alignments. This result shows that the plasmid is indeed integrated into the chromosome, rather than being a misassembly artifact (Tables S16 and S17).

## SUPPLEMENTAL MATERIAL

File S1 msystems.00479-26-s0001.txt Multiple sequence alignment.

Supplemental Figures msystems.00479-26-s0002.docx Figures S1–S6.

Supplemental Tables msystems.00479-26-s0003.xlsx Tables S1–S19.

ASM does not own the copyrights to Supplemental Material that may be linked to, or accessed through, an article. The authors have granted ASM a non-exclusive, world-wide license to publish the Supplemental Material files. Please contact the corresponding author directly for reuse.
