# Supplementary material for: Mobile genetic elements shape the evolution and adaptation of the marine Sulfitobacter genus
Source: mSystems. 2026 Jun 15;11(7):e00479-26. doi: 10.1128/msystems.00479-26 (PMC13386836; doi:10.1128/msystems.00479-26)
Supplement: Supplemental Figures — Figures S1–S6. [file msystems.00479-26-s0002.docx]

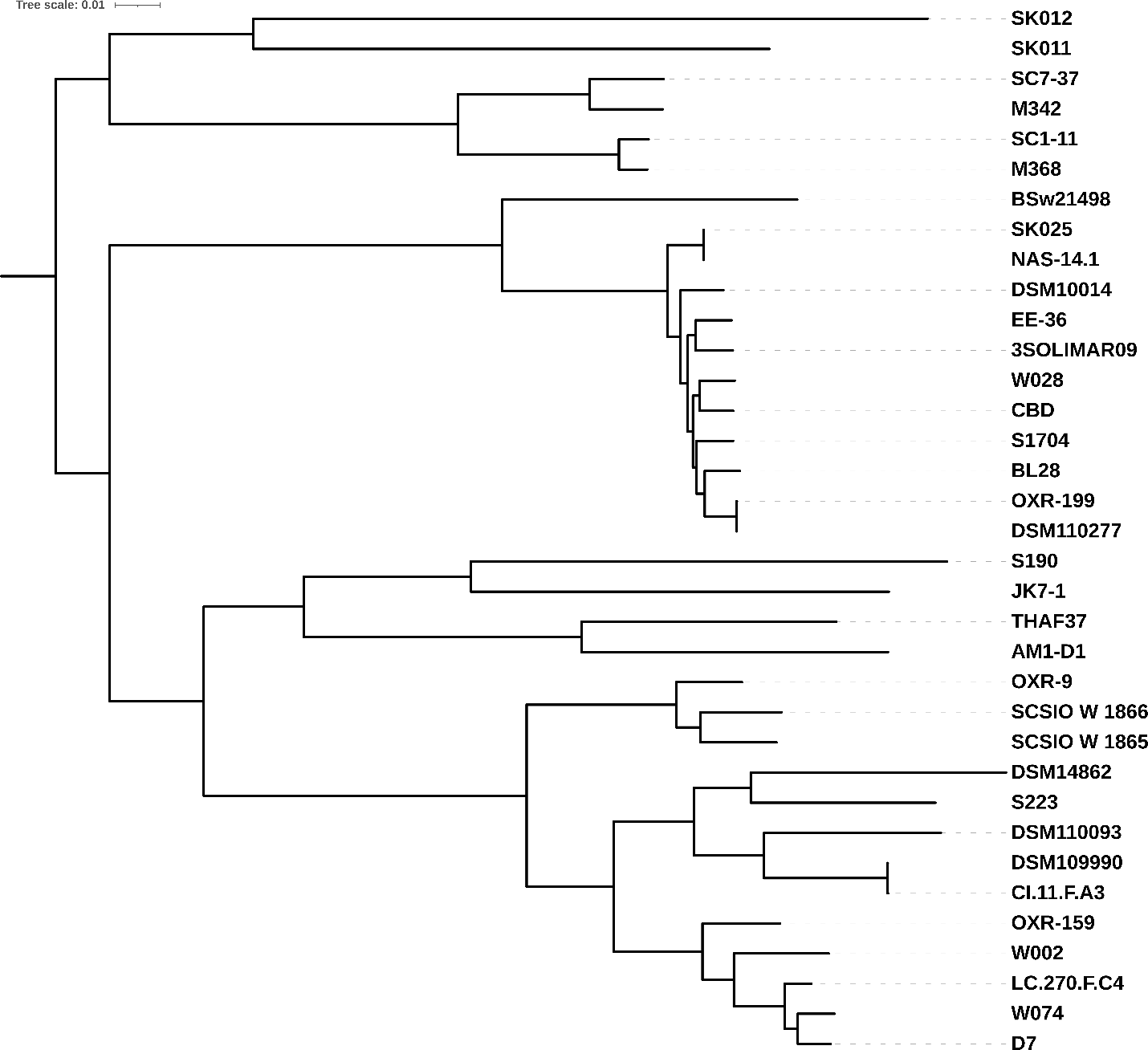


**Figure S1. Core genome phylogenetic tree of closed *Sulfitobacter* genera members.** This maximum-likelihood phylogenetic tree was constructed using concatenated alignments of single-copy core genes (n = 98) identified from 36 completely sequenced *Sulfitobacter* genomes. Core genes were defined based on ≥90% amino acid identity across genomes. The scale bar represents 0.01 substitutions per site, indicating genetic distance.


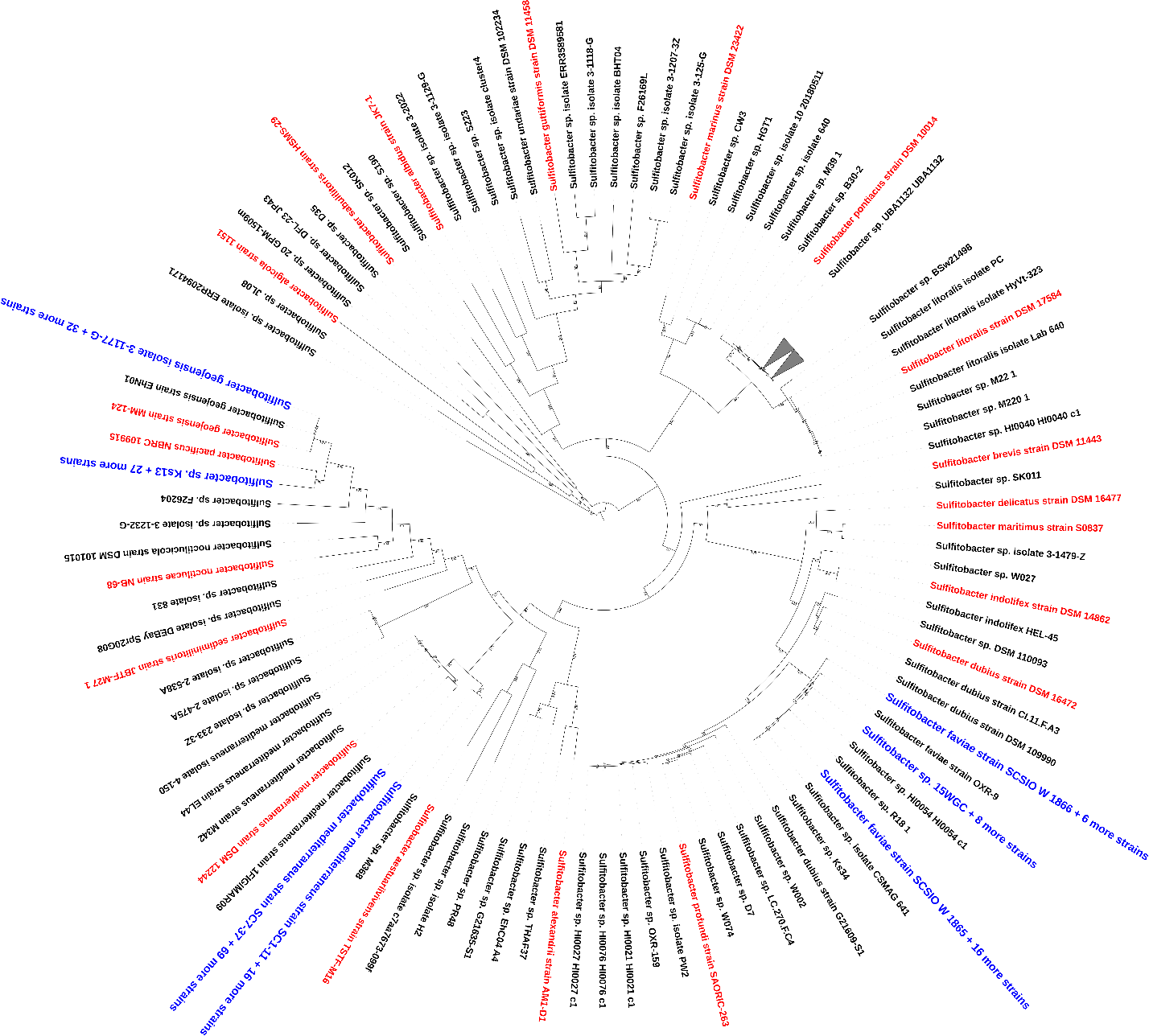


**Figure S2. Phylogenetic tree of *Sulfitobacter* genera genomes.** Maximum-likelihood phylogeny inferred from the concatenated multiple sequence alignment using IQ-TREE2 and visualized in iTOL. Genomes are grouped by species-level clades. Red branches indicate the type strain for each species; blue branches are representative strains selected from larger clades to reduce visual overcrowding while preserving the overall topology. The concatenated multiple sequence alignment (MSA) used for phylogenetic reconstruction is provided as Supplemental File 1. Accession numbers can be found in Table S19.


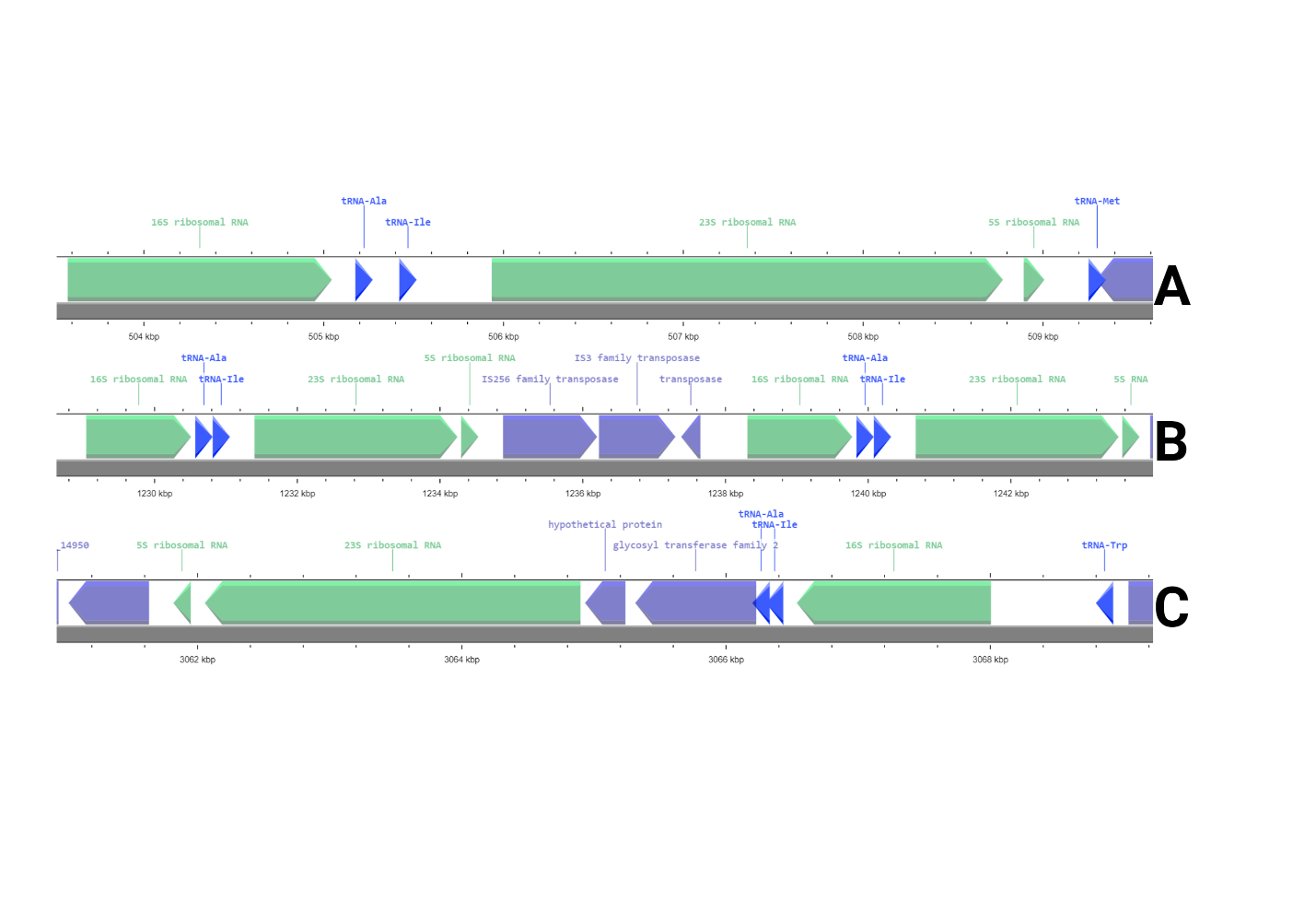


**Figure S3.** **Organization of the three *rrn* operons in *S. profundi* strain D7.** The genomic locations and structures of the three chromosomal ribosomal RNA (*rrn*) operons encoded in the D7 genome (NZ_CP020694.1).


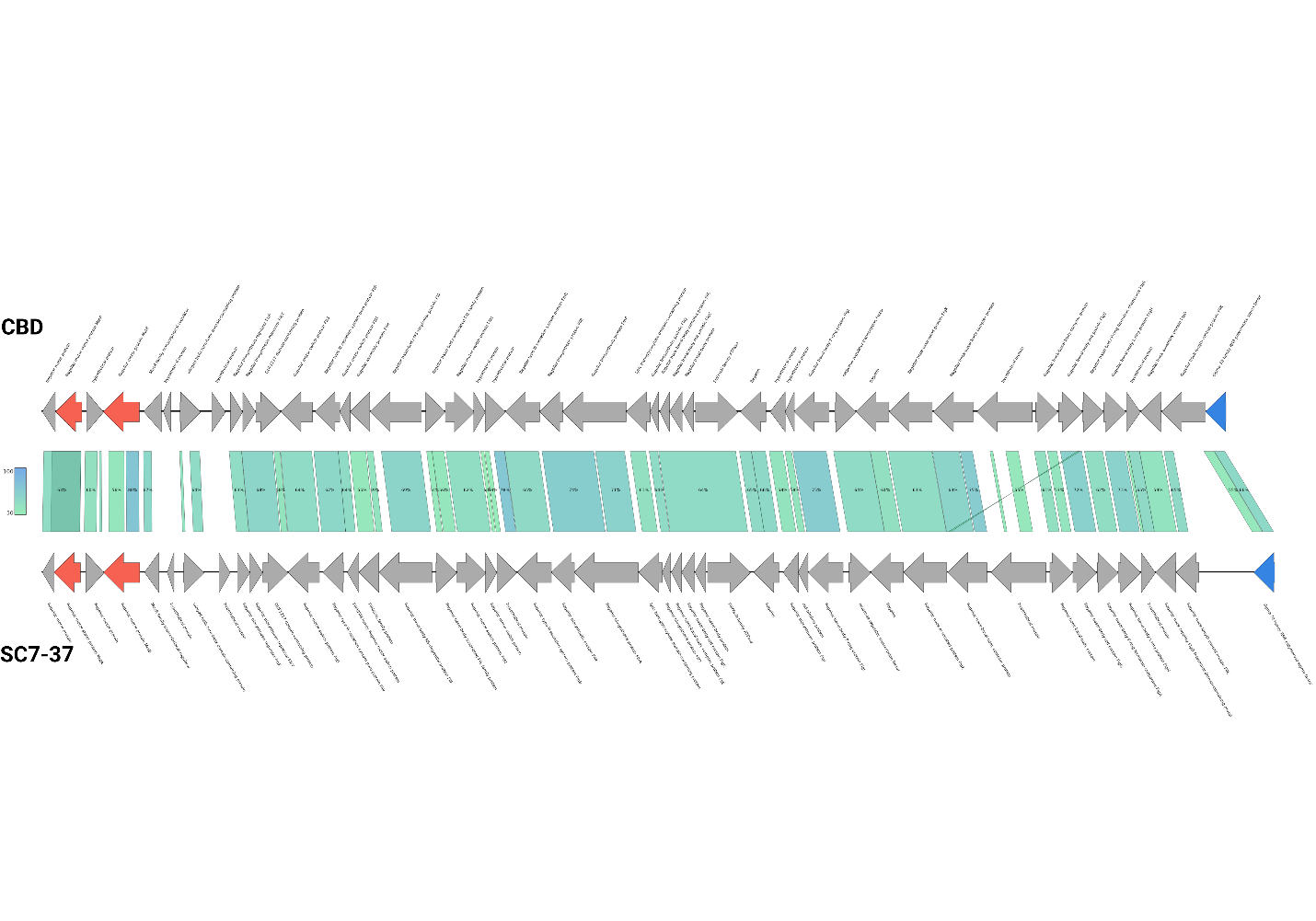


**Figure S4. Plasmid-encoded flagellar gene clusters in *S.* *pontiacus* strain CBD and *S. mediterraneus* SC7-37.** The plasmid-encoded flagellar biosynthetic gene clusters of strains CBD (NZ_CP072614.1)and SC7-37 (NZ_CP069000.1). Gene synteny and content illustrate conserved plasmid-mediated motility functions in distinct *Sulfitobacter* lineages.


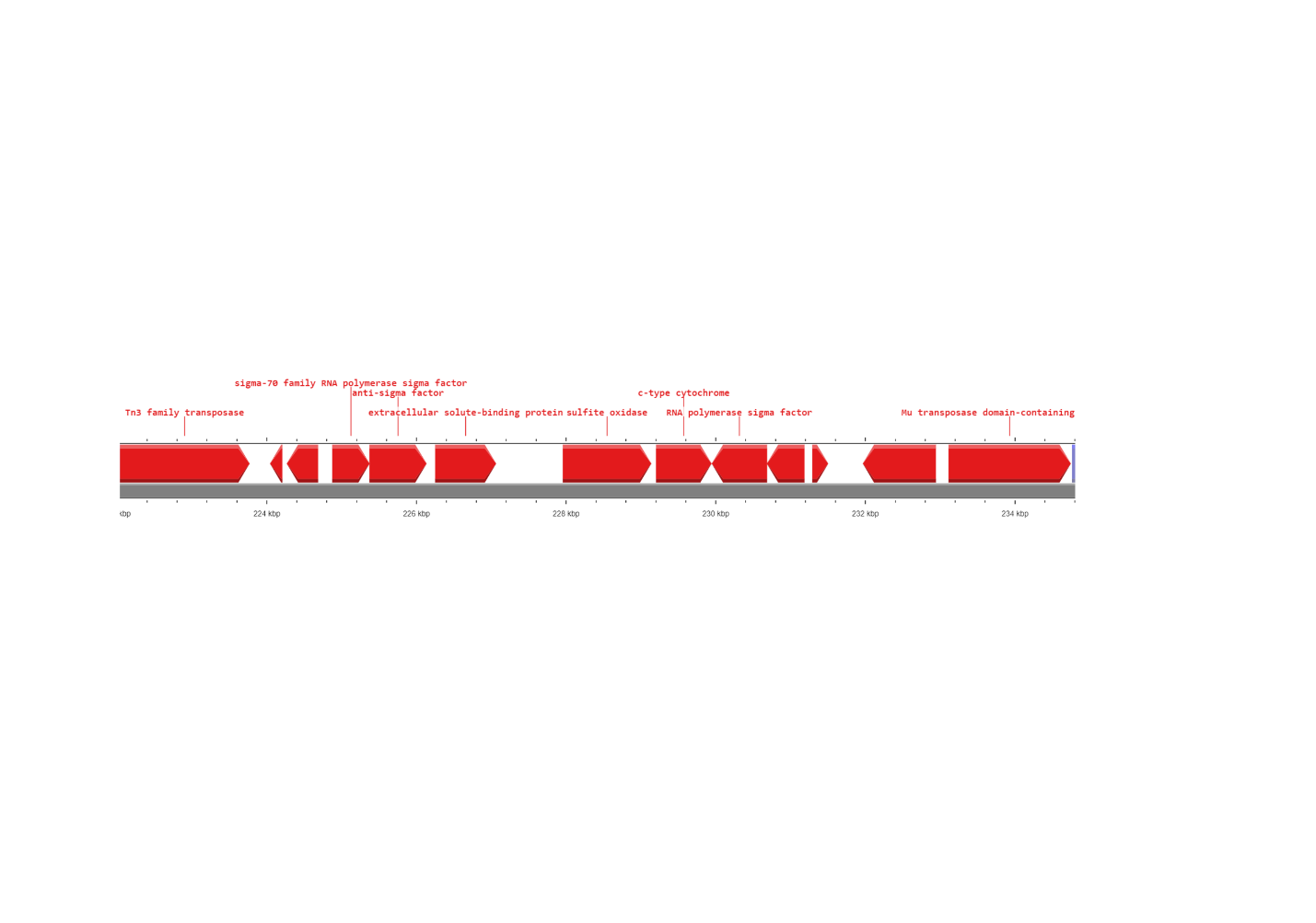


**Figure S5.** **Plasmid-encoded σ⁷⁰ family sigma factor in *S. pontiacus* DSM 110277.**
Genome organization of the σ⁷⁰ sigma factor gene located on the 239 kb plasmid (NZ_CP084960.1) is shown, along with surrounding genes. Genes without annotations are conserved hypothetical proteins.


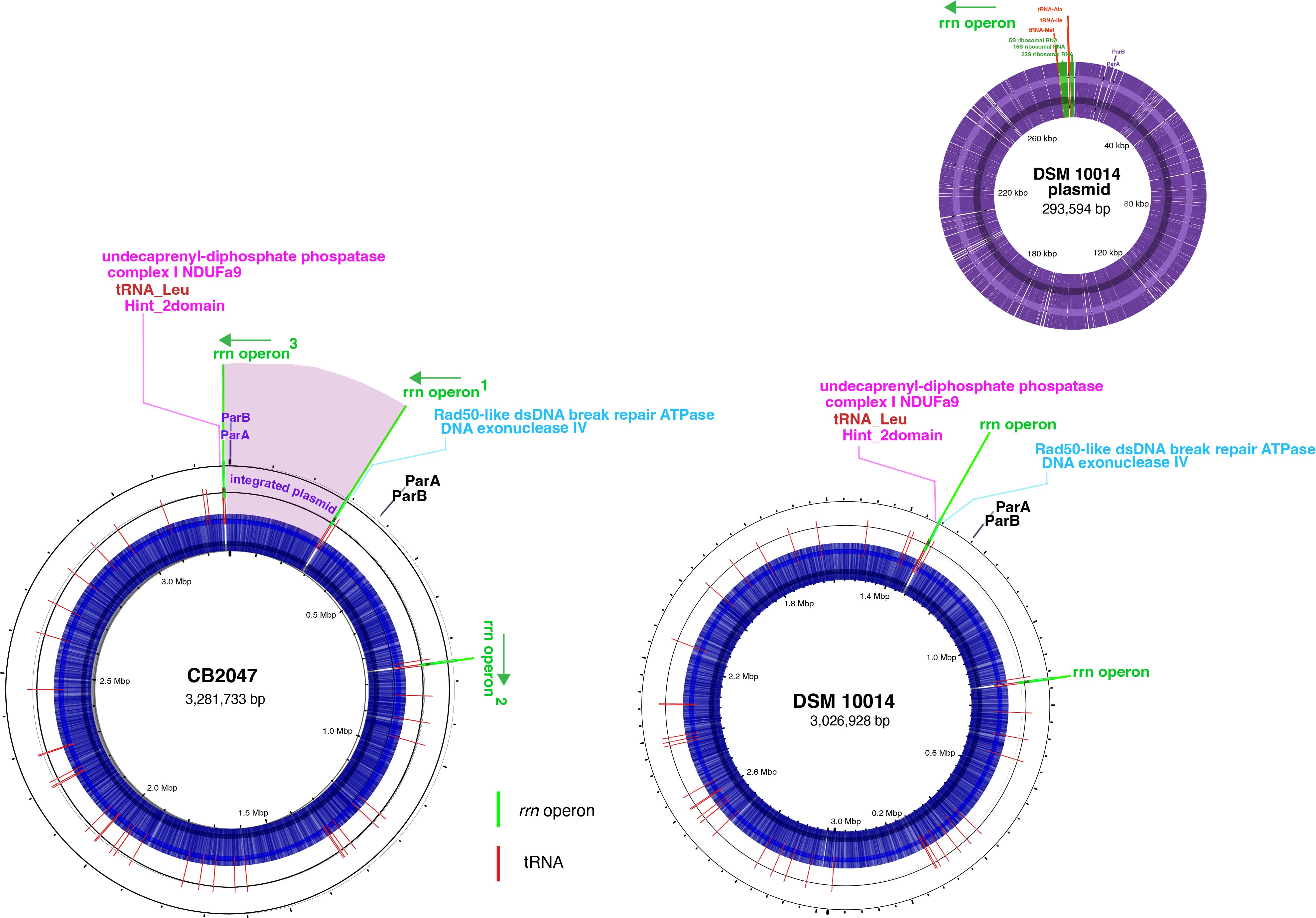


**Figure S6. Graphical representation of the integrated plasmid in *S. pontiacus* CB2047.** Strain DSM10014 is shown as a representative of the other *S. pontiacus* genomes in which the plasmid exists as an extrachromosomal element. In strain CB2047, 2 of the 3 *rrn* operons (*rrn*2: IV89_000702-706 and *rrn*3: IV89_003218-3214) are identical across the 5726 nucleotides of the operon. *rrn*1 (IV89_000274-IV89_000278) differs by 2 nucleotides within the gene encoding the 5S subunit, within the last 300 bp of the operon. IV89_000279-280, which encodes an AAA family ATPase and DNA exonuclease, respectively, are located 604 nucleotides upstream of the *rrn*1 operon. The near identity of the flanking *rrn* operons and absence of disrupted or mosaic operon structure are consistent with homologous recombination at an *rrn*-associated chromosomal site, resulting in integration of the plasmid while preserving an intact plasmid-encoded *rrn* operon.
